# Appraising the uptake and use of recommendations for a common outcome data set for clinical trials: a case study in fall injury prevention

**DOI:** 10.1186/s13063-016-1259-7

**Published:** 2016-03-10

**Authors:** Bethan Copsey, Sally Hopewell, Clemens Becker, Ian D. Cameron, Sarah E. Lamb

**Affiliations:** Oxford Clinical Trials Research Unit, Nuffield Department of Orthopaedics, Rheumatology and Musculoskeletal Sciences, University of Oxford, Oxford, UK; Department of Clinical Gerontology and Rehabilitation, Robert-Bosch-Hospital, Stuttgart, Germany; John Walsh Centre for Rehabilitation Research, University of Sydney, Kolling Institute, St Leonards, NSW Australia

**Keywords:** Core outcome set, Falls, Older adults

## Abstract

**Background:**

Many researchers and professional bodies are seeking consensus for core outcomes for clinical trials. The Prevention of Falls Network Europe (ProFaNE) developed a common outcome data set for fall injury prevention trials 10 years ago. This study assesses the impact of these recommendations.

**Methods:**

A systematic search (up to 16 January 2015) was performed using Web of Science, Scopus and PubMed for articles citing the ProFaNE recommendations. Randomised trials on fall prevention in older people were selected for further analysis. Data were extracted on study characteristics and adherence to the key domains recommended by the ProFaNE consensus: falls, fall injury, physical activity, psychological consequences and health-related quality of life. Details of non-recommended outcome measures used were also recorded.

**Results:**

The ProFaNE recommendations were cited in a total of 464 published articles, of which 34 were randomised trials on fall prevention in older people. Only one study (3 %) reported on all core domains. Most of the trials reported on falls (*n* = 32/34, 94 %) as a core outcome measure. Most of the recommendations within the falls domain were well-followed. Around half of the trials reported on fall-related injury (*n* = 16/34, 47 %). However, none reported the number of radiologically confirmed peripheral fracture events, which is the recommended outcome measure for injury. The other key domains (quality of life, physical activity and psychological consequences) were less frequently reported on, with a lack of consistency in the outcome measures used.

**Conclusions:**

The ProFaNE recommendations had a limited effect on standardising the reporting of outcomes in randomised trials on fall injury prevention in older people during the search period. Authors of consensus guidelines should consider maximising buy-in by including a diversity of geographic areas and academic disciplines at the development stage and using a solid dissemination strategy.

**Electronic supplementary material:**

The online version of this article (doi:10.1186/s13063-016-1259-7) contains supplementary material, which is available to authorized users.

## Background

Gaining consensus on the use of core outcomes for clinical trials is a major but fairly recent activity in many areas of medical research. This is in part due to the recent COMET (Core Outcome Measures in Effectiveness Trials) Initiative launched in 2010 which aimed to stimulate the development and application of core outcome sets [1, 2]. The COMET Initiative also states that consideration must be given to the uptake and implementation of a core outcome set [3]. Relatively few consensus guidelines have been evaluated in terms of impact to determine whether the underlying ethos of harmonising the selection and reporting of outcomes across trials can be achieved.

Five years prior to the launch of the COMET Initiative, the Prevention of Falls Network Europe (ProFaNE) outcome consensus group developed a core outcome set which provided recommendations for a common set of definitions and measures for clinical trials and meta-analyses for fall injury prevention [4]. The recommendations focus on five key domains: falls, fall injury, the psychological consequences of falling, health-related quality of life and physical activity. The domains provide a minimum set of outcomes which should be reported in clinical trials of fall injury prevention and, within each domain, the recommendation provides information on the outcome measures, definitions and methodology which should be used to assess the corresponding outcome.

The recommendations were developed by international expert consensus in three phases. First, experts in the field agreed on the key domains for outcome assessment during an international meeting. Second, systematic reviews were conducted to identify the quality and scope of the measures that were currently used in clinical trials. Finally, expert consensus recommendations for outcome measures in each domain were developed during a further 2-day international meeting and modified nominal group technique. The final recommendations suggested the domains that should be included in the data capture, and recommended measures for each domain.

If trials are all conducted and reported using a common set of elements, then the results of studies can be more effectively combined in systematic reviews and individual patient level meta-analyses of trials on fall injury prevention. As the number of randomised trials and systematic reviews in the area of falls and fall prevention has rapidly increased since the ProFaNE recommendations were published over 10 years ago, it should now be possible to evaluate their impact [5].

The aim of this study is to examine the uptake and use of the ProFaNE recommendations, specifically how often and under what circumstances it is cited. In addition, we explored whether the recommendations are used appropriately in a selection of randomised controlled trials on fall prevention in older people.

## Methods

### Sample selection

The Web of Science Citation Index, Scopus and PubMed databases were searched (up to 16 January 2015) to identify all articles citing the ProFaNE consensus recommendations paper since it was published in 2005 [4]. After removing duplicates, one reviewer (BC) screened the titles and abstracts of all retrieved reports to identify those that reported on a randomised controlled trial. We defined a randomised trial as a prospective study that assessed healthcare interventions in human participants who were randomly allocated to study groups. We obtained the full article for each record identified as a report of a randomised trial.

Article eligibility was further assessed using the full text articles. Articles were eligible if they reported the results of a randomised trial in the area of fall prevention in older people. A population was classified as older people if the trial excluded participants younger than 60 years of age, the mean age of the included participants was 60 years of age or above, or the target population was described as ‘older’, ‘elderly’, or ‘senior’. If multiple articles were published on the same trial, we included only the article that reported the primary outcome at the earliest post-intervention follow-up point analysed after trial completion.

### Data extraction

For all records citing the ProFaNE recommendations, we recorded the year of publication, publication type (e.g. randomised trial, observational study, methodological, editorial or review) and geographical region of the first author, using only the title and abstract.

For articles reporting the results of a randomised trial in the area of fall prevention in older people, we recorded the study characteristics of sample size, funding source, setting, intervention type and baseline characteristics of participants. If the baseline data were only presented by treatment group, only the values for the intervention group were extracted. Pilot data extraction was performed by two independent extractors (BC and SH) on a sample of five reports to ensure data relevance and accuracy, and consistency between extractors. As there were no major disagreements during pilot data extraction, data extraction for the remaining articles was performed by only one extractor (BC).

Data were also extracted on adherence to the ProFaNE recommendations (see Table 1). The key domains considered in the ProFaNE recommendations are falls, fall injury, physical activity, psychological consequences and health-related quality of life. We recorded whether each domain was reported in each article. When a domain was reported, we recorded the extent to which each of the recommended outcomes were reported and whether the recommended outcome measures and methods were used. Any non-recommended outcome measures used in any key domain were also recorded. We recorded whether any shortcomings of the core set of outcomes and outcome measures were discussed in each article, for example, any reason against using the recommended definition for a fall.

**Table 1:** Summary of recommendations from the Prevention of Falls Network Europe (ProFaNE) consensus

Recommendation 1: Domains and considerations
1. Domains should include falls, fall injury, physical activity, psychological consequences, and generic health-related quality of life (HRQoL)
2. The selection of measures should focus on community-dwelling populations
3. The common data set should consider cost and ease of application in a wide range of countries
4. The recommendations should include details on methods of measurement
5. The process (of developing a common data set) should be founded on a review of measures currently reported in clinical trials of fall and fall injury prevention interventions
Recommendation 2: Falls
1. A fall should be defined as ‘an unexpected event in which the participants come to rest on the ground, floor, or lower level’
2. Ascertainment must consider the lay perspective of falls. Participants should be asked, ‘In the past month, have you had any fall including a slip or trip in which you lost your balance and landed on the floor or ground or lower level?’
3. Falls should be recorded using prospective daily recording and a notification system with a minimum of monthly reporting. Telephone or face-to-face interview should be used to rectify missing data and to ascertain further details of falls and injuries
4. Fall data should be summarised as number of falls, number of fallers/non-fallers/frequent fallers, fall rate per person year, and time to first fall (as a safety measure)
5. Primary analysis of fall data should not be adjusted for physical activity, and reporting should include the absolute risk difference between groups
Recommendation 3: Injuries
1. The recommended common data set measure is the number of radiologically confirmed peripheral fracture events per person year. This should include the limbs and limb girdles
2. Injuries should be classified according to the International Classification of Diseases, 10th revision, classification system
3. Data should be collected prospectively, alongside and using the same methods as for fall reporting
4. Injury data should be summarised as peripheral fracture rate per person-year of follow-up, number of peripheral fractures, number of people sustaining peripheral fractures, and number of people sustaining multiple events
5. Primary analysis should not be adjusted for physical activity, and reporting should include the absolute risk difference between groups
Recommendation 4: Psychological consequences of falling
1. Psychological consequences of falls should be conceptualised in terms of fall-related self-efficacy, defined as ‘the degree of confidence a person has in performing common activities of daily living without falling’ and measured using the modified Falls Efficacy Scale (mFES)
2. The measure should be scored per published guidance
Recommendation 5: HRQoL
1. For the ProFaNE common outcome data set, the recommended measures of HRQoL are the Short Form 12 (SF-12) version 2 and European Quality of Life Instrument (EuroQoL EQ-5D)
Recommendation 6: Physical activity measures
1. Further research is required before a measure of physical activity can be recommended for inclusion in the common data set.
Recommendation 7: Time points for follow-up for the ProFaNE common data set
1. Many fall-prevention interventions require longer-term follow-up (12 months) because they have a delayed effect, taking time and compliance to evidence an effect

### Data analysis

Our main analysis focused on the number of reports of randomised trials in the area of fall prevention in older people that adhered to each recommendation in each domain.

We estimated the overall proportion of randomised trials in fall prevention in older people in a community-dwelling population published since 2005 that cited the ProFaNE recommendations using a denominator estimate of the number of randomised trials published after 2005 that were included in a Cochrane review of interventions for preventing falls in elderly people living in the community [5]. A crude estimate was then obtained by calculating the proportion of these trials that cited the ProFaNE recommendations. However, the Cochrane review only included trials published before 2012. We assumed the same rate of publication and extrapolated the approximate number of eligible trials since the recommendations were published, based on the average number of trials published per year from 1990 to 2012 which were included in the Cochrane review. This figure was used as a denominator to provide a second estimate of the proportion of randomised trials in the area of fall prevention in older people in a community-dwelling population that cited the ProFaNE recommendations.

## Results

### General characteristics of all articles citing the ProFaNE recommendations (*n* = 464)

**Fig. 1:**
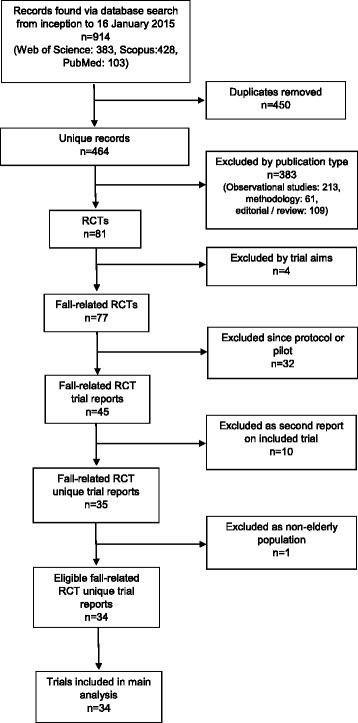
Identification of reports of randomised trials in the area of fall prevention among an elderly population

**Fig. 2:**
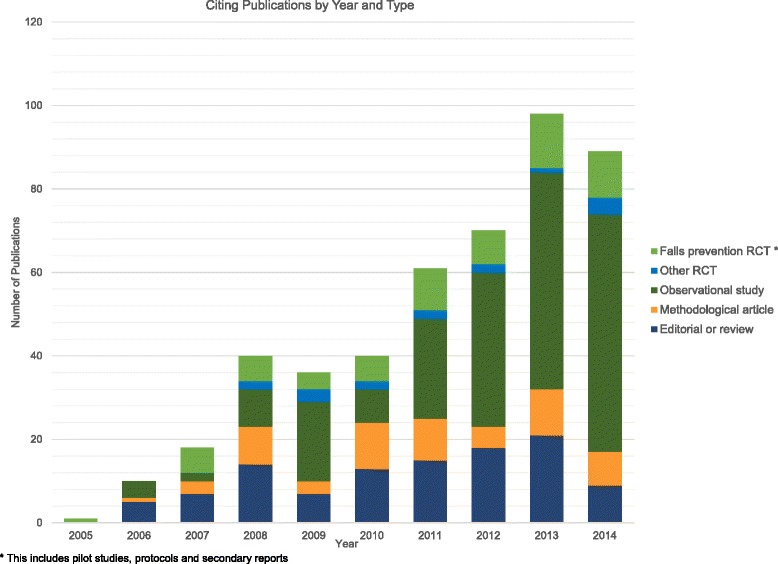
Number of publications citing the Prevention of Falls Network Europe (ProFaNE) recommendations by year and study type

We identified 464 articles that cited the ProFaNE recommendations and were published between October 2005 and January 2015 (Fig. 1). Half of these articles were from Europe (*n* = 237), of which 72/237 (16 %) were from the UK and Ireland. The majority of the non-European articles were from Australia and New Zealand (*n* = 107/464, 23 %) and North America (*n* = 82/464, 18 %). The majority of the articles citing the ProFaNE recommendations were observational studies (*n* = 213/464, 46 %), editorials or reviews (*n* = 109/464, 23 %) or methodological articles (*n* = 61/464, 13 %). Seventeen percent (*n* = 81/464) were reports of randomised controlled trials (Fig. 2).

Of the 81 articles reporting on randomised controlled trials, 34 reported the results of unique randomised trials in the area of fall prevention among an elderly population. These 34 articles were all included in our main analysis (Additional file 1). The main reasons for exclusion of reports on randomised controlled trials were that the reports were protocols (*n* = 26), pilot studies (*n* = 6), or a secondary report of an already included study (*n* = 10) (Fig. 1).

### General characteristics of randomised trials of fall prevention in older people (*n* = 34)

The majority of the 34 reports of randomised trials selected were published in specialist journals (*n* = 28/34, 82 %) and funded by non-industry sources (*n* = 24/34, 71 %). The trials assessed mainly interventions comprising several components (*n* = 16/34, 47 %), with many described as multi-factorial fall prevention programmes. Exercise was the most commonly examined single intervention (*n* = 14/34, 41 %). Most of the studies (*n* = 26/34, 76 %) were based in a community setting, with the remainder being based in hospitals (*n* = 5/34, 15 %) or assisted-living facilities (*n* = 3/34, 9 %) (see Table 2).

**Table 2:** Study characteristics of randomised trials of fall prevention in older adults citing Prevention of Falls Network Europe (ProFaNE) (*n* = 34)

Study characteristic	Number (%) if not otherwise stated
Number of centres	
Single centre	9 (26)
Multi-centre	16 (47)
Unclear	9 (26)
Number of treatment arms	
2 arms	26 (76)
3 or more arms	8 (24)
Funding source	
Industry	2 (6)
Non-industry	24 (71)
Both industry and non-industry	5 (15)
None required	1 (3)
Unclear	2 (3)
Types of intervention	
Multi-component	16 (47)
Exercise only	14 (41)
Advice or education only	2 (6)
Other single intervention	2 (6)
Population at high risk of falling (Yes)	18 (53)
Sample size (median (interquartile range))	233 (124, 401)
Proportion of female participants (min, max)	37 % to 100 %
Mean age of included participants (min, max)	62 to 88
Assessed falls as primary outcome	23 (68)
Measured primary outcome at ≥12 months	20 (59)

The median sample size of the trials was 233 (interquartile range = 124 to 401), ranging from 36 to 1314 participants per trial. Around half of the trials restricted their population to include only participants deemed at high risk of falling (*n* = 18/34, 53 %), based on their history of falls, mobility problems or associated medical conditions. Of those trials with populations restricted by risk of falling, two assessed people with dementia or cognitive impairment, three with Parkinson’s and stroke, and one with osteoporosis. The remaining trials did not restrict their population based on disease area.

Twenty-three of the 34 trials (68 %) used a primary outcome based on falls, such as fall rate, number of fallers or time to first fall. Other primary outcomes included physical performance measures (e.g. chair rise or balance score), bone mineral density, activities of daily living index and health-related quality of life measures. More than half (*n* = 20/34, 59 %) of the trials reported the primary outcome at a follow-up time point of 12 months or more.

The consensus was cited in the methods section of 24 (71 %) of the articles, most commonly referring to the use of recommended methodology, such as the definition of a ‘fall’, length of follow-up period or the procedures for recording and reporting falls.

### Adherence to recommendation 1: Domains and considerations (*n* = 34)

**Fig. 3:**
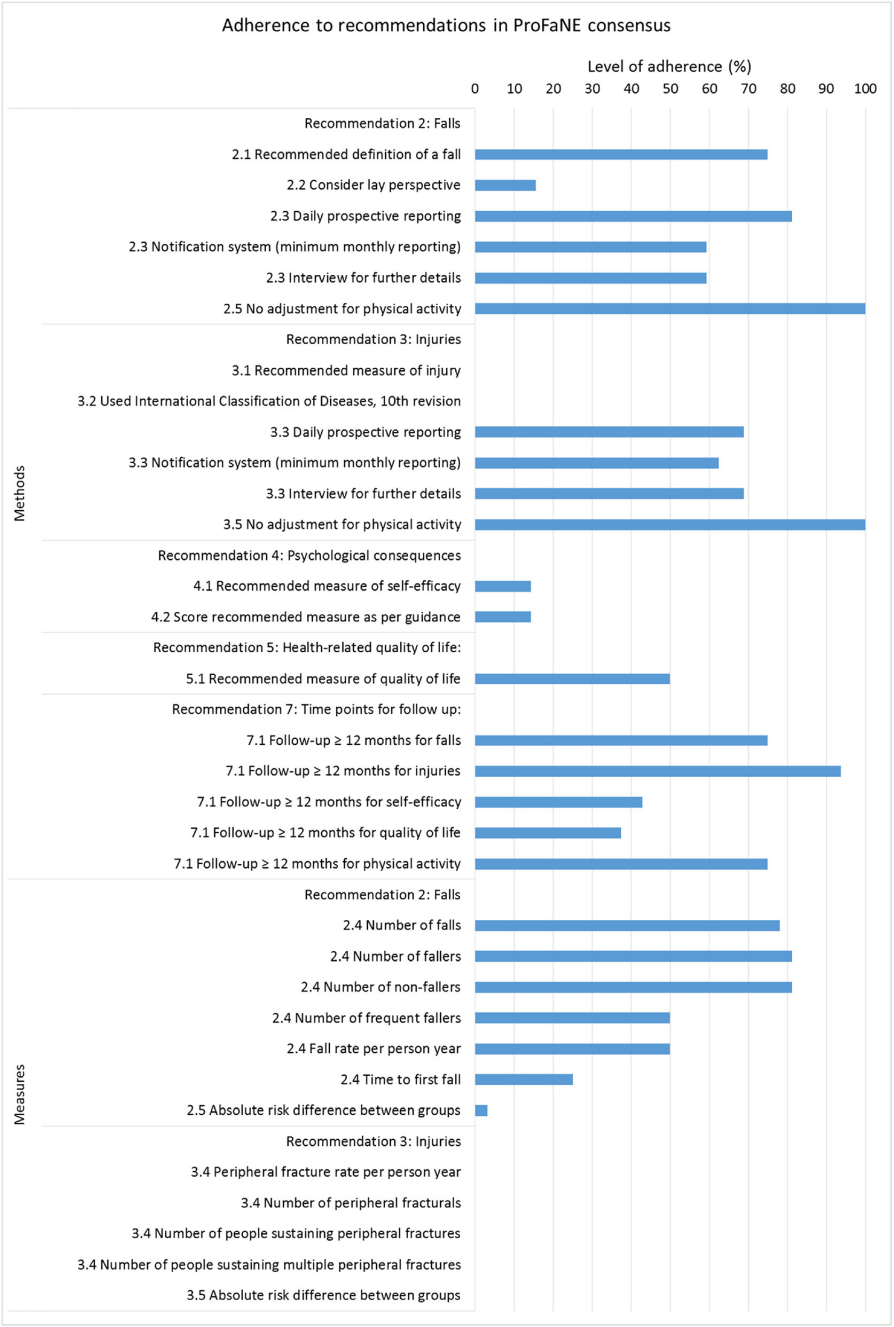
Level of adherence to the Prevention of Falls Network Europe (ProFaNE) recommendations

The most commonly reported domain was falls (*n* = 32/34, 94 %). Fall-related injury was the second most frequently reported domain (*n* = 16/34, 47 %). The other domains, psychological consequences of falling (*n* = 7/34, 21 %), health-related quality of life (*n* = 8/34, 24 %) and physical activity (*n* = 8/34, 24 %), were less frequently reported. All of the trials that reported at least one domain also reported the falls domain. Most of the trials reported on falls and one other domain (*n* = 14/34, 41 %). Only one study (3 %) reported all five of the recommended domains. Table 3 and Fig. 3 present a summary of the included trials’ adherence to each recommendation in each of the key domains.

**Table 3:** Adherence to the Prevention of Falls Network Europe (ProFaNE) recommendations

Recommendation 1: Domains and considerations (*n* = 34)	Yes – *n* (%)
1.1 Inclusion of domains	
Domains reported on:	
Falls	32 (94)
Fall injury	16 (47)
Psychological consequences	7 (21)
Health-related quality of life	8 (24)
Physical activity	8 (24)
Recommendation 2; Falls (*n* = 32)	Yes – *n* (%)
2.1 Recommended definition	
Defined a fall as ‘an unexpected event in which the participants come to rest on the ground, floor or lower level’	24 (75)
2.2 Lay perspective	
Considered lay perspective during ascertainment of information	5 (16)
Asked participants: ‘In the past month, have you had any fall including a slip or trip in which you lost your balance and landed on the floor or ground or lower level?’	0 (0)
2.3 Methods and systems for recording falls information	
Used daily prospective recording	26 (81)
Used a notification system with a minimum of monthly reporting	19 (59)
Used a telephone or face-to-face interview to rectify missing data and ascertain further details of falls	19 (59)
2.4 Summarising of fall data	
Reported number of falls	25 (78)
Reported number of fallers	26 (81)
Reported number of non-fallers	26 (81)
Reported number of frequent fallers	16 (50)
Reported fall rate per person year	16 (50)
Reported time to first fall	8 (25)
2.5 Covariate adjustment and further data summaries	
Did not adjust for physical activity in primary analysis	32 (100)
Reported absolute risk difference between groups	1 (3)
Recommendation 3: Injuries (*n* = 16)	Yes – *n* (%)
3.1 Recommended measure	
Reported number of radiologically confirmed peripheral fracture events per person year	0 (0)
3.2 Classification of injuries	
Used the International Classification of Diseases, 10th revision, classification system to classify injuries	0 (0)
3.3 Methods and systems for recording injury information	
Used daily prospective recording	11 (69)
Used a notification system with a minimum of monthly reporting	10 (63)
Used a telephone or face-to-face intervention to rectify missing data and ascertain further details of injuries	11 (69)
3.4 Summarising of injury data	
Reported peripheral fracture rate per person year of follow-up	0 (0)
Reported number of peripheral fractures	0 (0)
Reported number of people sustaining peripheral fractures	0 (0)
Reported number of people sustaining multiple peripheral fractures	0 (0)
3.5 Covariate adjustment and further data summaries	
Did not adjust for physical activity in primary analysis	16 (100)
Reported absolute risk difference between groups	0 (0)
Recommendation 4: Psychological consequences of falling (*n* = 7)	Yes – *n* (%)
4.1 Recommended measure	
Used the recommended modified Falls Efficacy Scale (mFES)	1 (14)
4.2 Scoring of measure	
Scored mFES as per published guidance	1 (14)
Recommendation 5: Health-related quality of life (*n* = 8)	Yes – *n* (%)
5.1 Recommended measure	
Used a recommended measure of health-related quality of life	4 (50)
Measured health-related quality of life using:	
Short Form 12 (SF-12)	1 (13)
European Quality of Life Instrument (EQ-5D)	3 (38)
Recommendation 6: Physical activity (*n* = 8)	Yes – *n* (%)
6.1 Outcome measure	
Used any measure of physical activity	8 (100)
Recommendation 7; Time points for follow-up	Yes – *n* (%)
7.1 Length of follow-up assessment	
Reported at follow-up of ≥12 months in domain of:	
Falls	24 (75)
Injuries	15 (94)
Psychological consequences of falling	3 (43)
Health-related quality of life	3 (38)
Physical activity	6 (75)

The recommendations were most frequently cited in the methods section of articles (*n* = 24), but were also cited in the introduction or background (*n* = 4) and discussion (*n* = 9)

### Adherence to recommendation 2: Falls (*n* = 32)

The falls domain recommendations had the highest level of adherence. The majority of the trials in the falls domain used the recommended definition of a fall (*n* = 24/32, 75 %). Alternative definitions of a fall were ‘a sudden unintentional change in position that caused an individual to land at a lower level, that is, on an object, the floor, or the ground, due to reasons other than sudden-onset paralysis, epileptic seizures, or overwhelming external forces’ and ‘any incident that brings a person down to the ground against their will’.

For data collection, daily prospective recording of falls (such as calendars or falls diaries) was common (*n* = 26/32, 81 %). Over half of the trials used a notification system with a minimum of monthly reporting (*n* = 19/32, 59 %) and a telephone or face-to-face interview to rectify missing data (*n* = 19/32, 59 %).

When summarising their results, most of the trials reported the number of falls (*n* = 25/32, 78 %), fallers (*n* = 26/32, 81 %) and non-fallers (*n* = 26/32, 81 %). Half of the trials reported the number of frequent fallers and fall rate per person year. None of the trials reported adjusting for physical activity in the primary analysis. The assessment of falls at 12 months’ or more follow-up was common (*n* = 24/32, 75 %). However, few of the trials reported the time to first fall (*n* = 8/32, 25 %), and only one trial (3 %) reported the absolute risk difference between groups as recommended by the consensus; instead, most of the trials (47 %) reported the incidence rate ratio (*n* = 15/32, 47 %).

### Adherence to recommendation 3: Fall-related injuries (*n* = 16)

The fall-related injury domain had the worst level of adherence. None of the trials used the recommended measure of the number of radiologically confirmed peripheral fracture events per person year. Instead, most of the trials reported combined fracture events (*n* = 7/16, 44 %), and some reported individual types of fractures, such spinal fractures (*n* = 2), facial fractures (*n* = 1) and head injuries (*n* = 1). The remaining trials considered fractures in addition to other serious events (e.g. concussion, loss of consciousness, wounds requiring suturing, dislocation or luxation). None of the trials reported using the recommended International Classification of Diseases classification system to classify injuries.

When fall-related injuries were reported, adherence to the recommendations on data collection methods was high. Around two thirds of the trials used daily prospective recording (*n* = 11/16, 69 %), a monthly notification system (*n* = 10/16, 63 %) and a follow-up interview for further information (*n* = 11/16, 69 %). Long-term follow-up assessment of fall-related injuries at 12 months or more was carried out in almost all of the trials (*n* = 15/16, 94 %).

### Adherence to recommendation 4: Psychological consequences of falling (*n* = 7)

Seven of the trials assessed the psychological consequences of falling in terms of fall-related self-efficacy. Only one of these seven trials used the recommended measure of the modified Falls Efficacy Scale (mFES). The rest used alternative versions of the falls efficacy scale (FES): the unmodified FES (*n* = 1), international FES (*n* = 3), shortened international mFES (*n* = 1) and Swedish version of the FES (*n* = 1). The psychological consequences of falling at 12 months’ or more follow-up were infrequently assessed (*n* = 3/7, 43 %).

Other trials reported on fear of falling in general (*n* = 5/34, 15 %) and balance confidence (*n* = 2/34, 6 %), rather than considering the effect of falling on confidence in carrying out daily living activities.

### Adherence to recommendation 5: Health-related quality of life (*n* = 8)

The domain of health-related quality of life was reported in eight of the trials. Half of these trials used the recommended measures Short Form 12 (SF-12) (*n* = 1/8, 13 %) or European Quality of Life Instrument (EQ-5D) (*n* = 3/8, 38 %). However, health-related quality of life in the long term was not frequently assessed (*n* = 3/8, 38 %).

### Adherence to recommendation 6: Physical activity (*n* = 8)

Physical activity was reported in eight of the trials. They all used different measures of physical activity: the Auckland Heart Study Physical Activity Questionnaire, an adaptation of the International Physical Activity Questionnaire, the Human Activity Profile, the LASA Physical Activity Questionnaire, a modified version of the Physical Activity Questionnaire for the Elderly, the Phone-FITT household physical activity levels, piezo-electric accelerometry (tracking activity by measuring motion) and a self-developed questionnaire on frequency and duration of exercise. Most of these trials (*n* = 6/8, 75 %) also reported a follow-up assessment of physical activity at 12 months’ or more follow-up.

### Assessing the level of impact as part of the wider research area

We examined the Cochrane review of fall prevention interventions in community settings [5]. Seventy-three of the included randomised trials were published between 2006 and 2011, with an average of 12 (standard deviation of 5) falls-related trials published per year. Only 15 (21 %) of these trials cited the ProFaNE recommendations, with an increase in the proportion of trials citing the ProFANE recommendations over time, ranging from 17 % (3 of 18 studies) in the period 2006 to 2007 to 25 % (8 of 32 studies) in the period 2010 to 2011.

Extrapolating from the calculations above and taking into account that more randomised trials are being published each year, we estimated a publishing rate of 20 trials per year, or 180 falls-related trials published between 2006 and 2014. We found that 26 trials on community-dwelling populations cited the ProFaNE recommendations in this period. A conservative estimate of the proportion of falls-related trials that cite the ProFaNE recommendations is, therefore, 14 %. The awareness of the ProFaNE recommendations can be conservatively summarised as being used in approximately one seventh of all falls-related randomised trials in community-dwelling older people.

## Discussion

### Summary of main findings

This study aimed to assess the impact of the ProFaNE recommendations for core outcome measures, particularly within randomised trials of fall injury prevention in older people, and found that the recommendations have had a limited impact. Although adherence to the recommendations was poor in some domains, the level of adherence was better within the domain of falls, which is the primary outcome for most trials in this field. The number of studies citing the recommendations has increased over time and by geographical region. It is likely that this trend will continue, as increased usage leads to knowledge of the recommendations becoming more widespread and improved practices in fall prevention research as a whole.

A key issue is lack of consistency in adherence to the recommendations. The recommendations in the falls domain were generally well-followed; most of the examined trials reported using the recommended methods and measures, such as the definition of a fall, daily prospective recording and summarising results by the number of falls, fallers and frequent fallers. Although it is important to note that the recommended definition of a fall and very similar definitions were in use prior to the ProFaNE initiative [6]. However, the majority of the randomised trials of fall prevention did not consider the domains of self-efficacy, quality of life or physical activity. Where these domains were reported, the level of adherence to the corresponding recommendations was poor. Although adherence varied by trial and domain, some studies followed almost all recommendations for the domains on which they reported; most notably Elley et al. reported on all five recommended domains and achieved the equal highest level of adherence of the included studies in each of the domains except the falls domain where adherence remained above average (Additional file 2) [7]. Only two trials did not report on the falls domain; Vaapio et al. only reported on health-related quality of life and Schepens et al. did not report on any of the recommended domains, instead assessing effectiveness of a fall prevention intervention by examining uptake of specific fall prevention behaviours and evaluating knowledge of fall threats [8, 9].

Only one study used the recommended measure of the mFES. However, the ProFaNE consensus acknowledges the need for further modification of this measure. Several studies used outcome measures for self-efficacy which were developed after the ProFaNE recommendations were published. Indeed, four trials used the FES-I which was developed to address the deficiencies of the mFES highlighted by the ProFaNE consensus [10].

Although only 8 of the 34 trials reported on health-related quality of life, half of these trials used a recommended measure, which is encouraging. However, further increases in the use of the recommended health-related quality of life measures are needed to facilitate health economic analysis and to capture the broader effects of fall prevention intervention, which could be quite substantial.

Around half of the randomised trials of fall prevention assessed fall-related injuries, yet none used the recommended measure or classification system of injuries, or presented the recommended summary of results. However, a possible cause for the low adherence to recommendations in the injury domain is that the corresponding systematic review was not published until 2012 [11]. In contrast, the review on the falls domain was published in 2006 and has been widely cited, thus increasing awareness [12]. Adherence to the recommendations on injury may, therefore, increase in future with the rise in supporting evidence. It is also possible that changes in injury pattern, and of access to more robust routine data means that this area of the consensus needs to be updated.

This study also found that fall prevention trials used measures of physical activity that were not considered in the original consensus, such as the Human Activity Profile Test [13]. The original ProFaNE recommendations also stimulated the development of the Phone-FITT measure, which was used by one study [14].

In addition, the increase in the number of observational studies citing the recommendations is notable; however, since the recommendations were developed for randomised controlled trials, adherence to the recommendations in non-randomised studies has not be considered in this study.

### Strengths and limitations

This study is one of the first to assess the impact of a recommendation for a core set of outcomes [1, 15, 16]. It includes valuable information on the types of studies that cite the recommendations, where these studies originate from, and how the citation pattern has changed over time. Several databases were searched to identify publications that cited the recommendations, and eligibility was not restricted by language.

The results are limited because impact has only been assessed using publications that cited the ProFaNE recommendations. It is possible that the true impact of the recommendations is greater than estimated. Some trials may have used the recommendations without citing the corresponding article or may have cited the corresponding systematic reviews that formed the basis of the recommendations [11, 12, 17, 18]. Likewise, adherence to the recommendations was assessed based on the information reported in each publication. Although this is a reasonable approximation, it may not reflect current practice. Trials may follow recommendations without explicitly referring to them. Moreover, trials published in the period immediately following the publication of the recommendations may not have had sufficient time to enact any changes to their methodology. A more accurate estimation of the uptake of the recommendations could be produced by conducting a systematic review of randomised trials to compare practice prior to and following publication of the recommendations, akin to Bautista-Molano et al. [15].

A further limitation is that the analysis was restricted to the earliest article published on the trial which reporting post-intervention results for the primary outcome. This could result in underestimation of the proportion of articles reporting certain domains since secondary outcomes and longer-term follow-up are sometimes reported in separate publications.

Methods for estimating the level of impact were limited due to difficulties in finding an appropriate denominator. Using the Cochrane review in the area allowed us to approximate the proportion of studies citing the ProFaNE guidance; yet this was limited as the included studies were only published up to 2012. Hence, as this study aimed to assess impact until 2015, this estimate may not be accurate due to the variation in publication rates over time. In addition, as this is one of the first studies to evaluate the impact of a core outcome set, the relative impact compared to other recommendations is unknown.

### Implications for updating the ProFaNE recommendations

Our study suggests that little adjustment is required to the recommendations in the falls domain. Few of the examined trials reported considering the lay perspective when ascertaining information on falls. However, it seems likely that this was due to lack of reporting, not poor practice, as none of the trials reported the specific phrasing they used when questioning participants on prior falls.

Poor and inconsistent adherence to the other domains highlights that improvements can be made. The recommendations in the injury domain most urgently require improvement. The recommendations that coincide with those in the general falls domain had high levels of adherence, but those specific to fall-related injuries were not followed. Most trials considered any fracture resulting from a fall that was reported by a participant. None of the studies reported using radiological confirmation, categorising by the International Classification of Diseases or considering peripheral fractures separately. The ProFaNE recommendations can be improved by expanding the set of injuries considered beyond peripheral fractures, such as by including head injuries or vertebral fractures. It is possible that the International Classification of Diseases classification is considered too burdensome for data collection. A simplified approach to classification may encourage the presentation of different types of injuries separately, such as the system for categorisation proposed by Schwenk and colleagues based on symptoms and medical care use [11].

The findings confirm deficiencies discussed in the original recommendations. The recommended measure for assessing psychological consequences of falling via self-efficacy must be clarified. In light of this, it is planned to review and potentially update the recommendations.

The ProFaNE consensus has motivated further research into the methodology and conduct of fall prevention trials, such as the development of new outcome measures and the ProFaNE taxonomy for classification of fall prevention interventions [10, 14, 19]. Any update of the ProFaNE guidance should also involve consideration of additional instruments for measuring exposure to physical activity for inclusion in the core outcome set, such as the more recently developed Phone-FITT [14].

### Issues to consider when developing and disseminating future recommendations for core outcomes

Overall, evaluating the impact of the ProFaNE recommendations has been a useful process. Evaluation through a review of uptake and usage can show how the core outcome set is being used in practice. It has highlighted key areas where recommendations were not being followed, indicating that future update or clarification is needed. Regular evaluation of the impact can provide evidence of deficiencies in the core outcome set to inform discussion about whether the recommendations are current and fit-for-purpose.

As stated by the COMET Initiative, there should not only be a focus on the development of core outcome sets, but also consideration to methods for dissemination. Several studies (7 of the 34 randomised trials on fall prevention in older people) included an author who was involved in the development of the ProFaNE recommendations. In addition, the proportion of studies citing the ProFaNE recommendations was much greater in regions where members of the outcome consensus group are based, estimated as 16 % in UK and Europe and 12 % in Australia and New Zealand compared to 7 % in Asia and 0 % elsewhere. Therefore, the involvement of an international and diverse group when developing a set of core outcomes measures can not only increase the applicability of recommendations but could potentially increase impact through improved reach. When creating recommendations for core outcome measures, strategies to maximise inclusivity at the time of development could be beneficial in terms of increased usage and wider dissemination. Moreover, dissemination of future recommendations for core outcome measures is likely to be greater and faster, due to increased usage and availability of modern communication technology. For example, social media can allow authors to rapidly increase awareness which could be used to improve dissemination, as well as involvement in development.

## Conclusions

The ProFaNE recommendations have had a limited effect on harmonising the reporting of outcomes in randomised trials on fall prevention, and has stimulated further methodological developments [10, 14, 19]. There have been improvements in the reporting of falls, but poor improvement in other domains. Recommendations in the domains of fall-related injuries and self-efficacy have been poorly followed. Future updates of the recommendations should focus on these areas and should incorporate more recent evidence by considering newly developed outcome measures and methodology [10, 14, 19]. Authors of consensus guidelines should consider maximising buy-in by including a diversity of geographic areas and academic disciplines at the development stage and using a solid dissemination strategy.

## Additional files

Additional file 1: Included and excluded randomised trials. (PDF 218 kb) Additional file 2: Table S1. Level of adherence to recommendations within domains by trial. (PDF 229 kb)

## Abbreviations

COMET: Core Outcome Measures in Effectiveness Trials
FES: Falls Efficacy Scale
HRQoL: health-related quality of life
mFES: modified Falls Efficacy Scale
ProFaNE: Prevention of Falls Network Europe
