# Supplementary material for: Appraising the uptake and use of recommendations for a common outcome data set for clinical trials: a case study in fall injury prevention
Source: Trials. 2016 Mar 10;17:131. doi: 10.1186/s13063-016-1259-7 (PMC4785736; doi:10.1186/s13063-016-1259-7)
Supplement: Additional file 1: — Included and excluded randomised trials. (PDF 218 kb) [file 13063_2016_1259_MOESM1_ESM.pdf]

Additional file 1: Included and excluded randomised trials

INCLUDED RANDOMISED TRIALS

1. Batchelor FA, Hill KD, Mackintosh SF, Said CM, Whitehead CH. Effects of a multifactorial falls prevention program for people with stroke returning home after rehabilitation: a randomized controlled trial. Arch Phys Med Rehabil. 2012;93(9):1648-55.
2. Duque G, Boersma D, Loza-Diaz G, Hassan S, Suarez H, Geisinger D, et al. Effects of balance training using a virtual-reality system in older fallers. Clin Interv Aging. 2013;8:257-63.
3. Elley CR, Robertson MC, Garrett S, Kerse NM, McKinlay E, Lawton B, et al. Effectiveness of a falls-and-fracture nurse coordinator to reduce falls: a randomized, controlled trial of at-risk older adults. J Am Geriatr Soc. 2008;56(8):1383-9.
4. Ferrer A, Formiga F, Sanz H, de Vries OJ, Badia T, Pujol R. Multifactorial assessment and targeted intervention to reduce falls among the oldest-old: a randomized controlled trial. Clin Interv Aging. 2014;9:383-94.
5. Fitzharris MP, Day L, Lord SR, Gordon I, Fildes B. The Whitehorse NoFalls trial: effects on fall rates and injurious fall rates. Age Ageing. 2010;39(6):728-33.
6. Freiberger E, Menz HB, Abu-Omar K, Rutten A. Preventing falls in physically active community-dwelling older people: a comparison of two intervention techniques. Gerontology. 2007;53(5):298-305.
7. Goodwin VA, Richards SH, Henley W, Ewings P, Taylor AH, Campbell JL. An exercise intervention to prevent falls in people with Parkinson's disease: a pragmatic randomised controlled trial. J Neurol Neurosurgery Psychiatry. 2011;82(11):1232-8.
8. Hill AM, Hoffmann A, McPhail S, Beer C, Hill KD, Oliver D, et al. Evaluation of the sustained effect of inpatient falls prevention education and predictors of falls after hospital discharge-follow-up to a randomized controlled trial. J Gerontol A Biol Sci Med Sci. 2011;66 A(9):1001-12.

9. Kemmler W, Von Stengel S, Engelke K, Häberle L, Kalender WA. Exercise effects on bone mineral density, falls, coronary risk factors, and health care costs in older women: the randomized controlled senior fitness and prevention (SEFIP) study. Arch Intern Med. 2010;170(2):179-85.

10. Kovacs E, Jonasne IS, Karoczi CK, Korpos A, Gondos T. Effects of a multimodal exercise program on balance, functional mobility and fall risk in older adults with cognitive impairment: a randomized controlled single-blind study. Eur J Phys Rehabil Med. 2013;49(5):639-48.

11. Kovacs E, Prokai L, Meszaros L, Gondos T. Adapted physical activity is beneficial on balance, functional mobility, quality of life and fall risk in community-dwelling older women: a randomized single-blinded controlled trial. Eur J Phys Rehabil Med. 2013;49(3):301-10.

12. Logan PA, Coupland CAC, Gladman JRF, Sahota O, Stoner-Hobbs V, Robertson K, et al. Community falls prevention for people who call an emergency ambulance after a fall: randomised controlled trial. BMJ. 2010;340.

13. Luukinen H, Lehtola S, Jokelainen J, Väänänen-Sainio R, Lotvonen S, Koistinen P. Pragmatic exercise-oriented prevention of falls among the elderly: a population-based, randomized, controlled trial. Prev Med. 2007;44(3):265-71.

14. McMurdo MET, Price RJG, Shields M, Potter J, Stott DJ. Should oral nutritional supplementation be given to undernourished older people upon hospital discharge? A controlled trial. J Am Geriatr Soc. 2009;57(12):2239-45.

15. Palvanen M, Kannus P, Piirtola M, Niemi S, Parkkari J, Järvinen M. Effectiveness of the chaos falls clinic in preventing falls and injuries of home-dwelling older adults: a randomised controlled trial. Injury. 2014;45(1):265-71.

16. Perula LA, Varas-Fabra F, Rodriguez V, Ruiz-Moral R, Fernandez JA, Gonzalez J, et al. Effectiveness of a multifactorial intervention program to reduce falls incidence among community-living older adults: a randomized controlled trial. Arch Phys Med Rehabil. 2012;93(10):1677-84.

17. Rapp K, Lamb SE, Büchele G, Lall R, Lindemann U, Becker C. Prevention of falls in nursing homes: subgroup analyses of a randomized fall prevention trial. J Am Geriatr Soc. 2008;56(6):1092-7.

18. Schepens SL, Panzer V, Goldberg A. Randomized controlled trial comparing tailoring methods of multimedia-based fall prevention education for community-dwelling older adults. Am J Occup Ther. 2011;65(6):702-9.

19. Shigematsu R, Okura T, Nakagaichi M, Tanaka K, Sakai T, Kitazumi S, et al. Square-stepping exercise and fall risk factors in older adults: a single-blind, randomized controlled trial. J Gerontol A Bio Sci Med Sci. 2008;63(1):76-82.

20. Smulders E, Weerdesteyn V, Groen BE, Duysens J, Eijsbouts A, Laan R, et al. Efficacy of a short multidisciplinary falls prevention program for elderly persons with osteoporosis and a fall history: a randomized controlled trial. Arch Phys Med Rehabil. 2010;91(11):1705-11.

21. Spink MJ, Menz HB, Fotoohabadi MR, Wee E, Landorf KB, Hill KD, et al. Effectiveness of a multifaceted podiatry intervention to prevent falls in community dwelling older people with disabling foot pain: randomised controlled trial. BMJ. 2011;342.

22. Taylor D, Hale L, Schluter P, Waters DL, Binns EE, McCracken H, et al. Effectiveness of tai chi as a community-based falls prevention intervention: a randomized controlled trial. J Am Geriatr Soc. 2012;60(5):841-8.

23. Tilson JK, Wu SS, Cen SY, Feng QS, Rose DR, Behrman AL, et al. Characterizing and identifying risk for falls in the leaps study a randomized clinical trial of interventions to improve walking poststroke. Stroke. 2012;43(2):446-52.

24. Trombetti A, Hars M, Herrmann FR, Kressig RW, Ferrari S, Rizzoli R. Effect of music-based multitask training on gait, balance, and fall risk in elderly people a randomized controlled trial. Arch Intern Med. 2011;171(6):525-33.

25. Vaapio S, Salminen M, Vahlberg T, Sjosten N, Isoaho R, Aarnio P, et al. Effects of risk-based multifactorial fall prevention on health-related quality of life among the community-dwelling aged: a randomized controlled trial. Health Qual Life Outcomes. 2007;5:20.

26. van Het Reve E, de Bruin ED. Strength-balance supplemented with computerized cognitive training to improve dual task gait and divided attention in older adults: a multicenter randomized-controlled trial. BMC Geriatr. 2014;14(1):134.

27. Vind AB, Andersen HE, Pedersen KD, Jorgensen T, Schwarz P. An outpatient multifactorial falls prevention intervention does not reduce falls in high-risk elderly Danes. J Am Geriatr Soc. 2009;57(6):971-7.

28. Von Stengel S, Kemmler W, Engelke K, Kalender WA. Effects of whole body vibration on bone mineral density and falls: results of the randomized controlled ELVIS study with postmenopausal women. Osteoporos Int. 2011;22(1):317-25.

29. Voukelatos A, Cumming RG, Lord SR, Rissel C. A randomized, controlled trial of tai chi for the prevention of falls: the central sydney tai chi trial. J Am Geriatr Soc. 2007;55(8):1185-91.

30. Zieschang T, Schwenk M, Oster P, Hauer K. Sustainability of motor training effects in older people with dementia. J Alzheimers Dis. 2013;34(1):191-202.

31. Faes MC, Reelick MF, Melis RJ, Borm GF, Esselink RA, Olde Rikkert MG. Multifactorial fall prevention for pairs of frail community-dwelling older fallers and their informal caregivers: a dead end for complex interventions in the frailest fallers. J Am Med Dir Assoc. 2011;12(6):451-8.

32. Moller UO, Kristensson J, Midlov P, Ekdahl C, Jakobsson U. Effects of a one-year home-based case management intervention on falls in older people: a randomized controlled trial. J Aging Phys Act. 2014;22(4):457-64.

33. Shigematsu R, Okura T, Sakai T, Rantanen T. Square-stepping exercise versus strength and balance training for fall risk factors. Aging Clin Exp Res. 2008;20(1):19-24.

34. Pérez-Ros P, Martínez-Arnau F, Tormos Miñana I, López Aracil A, Oltra Sanchis MC, Pechene Mera LE, et al. Preliminary results of a community fall prevention programme: Precan study (falls prevention in La Ribera). Rev Esp Geriatr Gerontol. 2014;49(4):179-83.

EXCLUDED RANDOMISED TRIALS

Excluded since protocol or pilot study

1. Asmidawati A, Hamid TA, Hussain RM, Hill KD. Home based exercise to improve turning and mobility performance among community dwelling older adults: protocol for a randomized controlled trial. BMC Geriatr. 2014;14(1).
2. Barker KL, Javaid MK, Newman M, Lowe CM, Stallard N, Campbell H, et al. Physiotherapy Rehabilitation for Osteoporotic Vertebral Fracture (PROVE): study protocol for a randomised controlled trial. Trials. 2014;15.
3. Batchelor FA, Hill KD, Mackintosh SF, Said CM, Whitehead CH. The FLASSH study: protocol for a randomised controlled trial evaluating falls prevention after stroke and two sub-studies. BMC Neurol. 2009;9:14.
4. Blank WA, Freiberger E, Siegrist M, Landendoerfer P, Linde K, Schuster T, et al. An interdisciplinary intervention to prevent falls in community-dwelling elderly persons: protocol of a cluster-randomized trial PreFalls. BMC Geriatr. 2011;11.
5. Cabral KD, Perracini MR, Soares AT, Stein FD, Sera CTN, Tiedemann A, et al. Effectiveness of a multifactorial falls prevention program in community-dwelling older people when compared to usual care: study protocol for a randomised controlled trial (Prevquedas Brazil). BMC Geriatr. 2013;13.
6. Campbell AJ, Robertson MC, La Grow SJ, Kerse NM, Sanderson GF, Jacobs RJ, et al. Randomised controlled trial of prevention of falls in people aged >= 75 with severe visual impairment: the VIP trial. BMJ. 2005;331(7520):817-20A.
7. Close JCT, Wesson J, Sherrington C, Hill KD, Kurrle S, Lord SR, et al. Can a tailored exercise and home hazard reduction program reduce the rate of falls in community dwelling older people with cognitive impairment: protocol paper for the i-FOCIS randomised controlled trial. BMC Geriatr. 2014;14(1).

8. Cockayne S, Adamson J, Martin BC, Fairhurst C, Hewitt C, Hicks K, et al. The REFORM study protocol: a cohort randomised controlled trial of a multifaceted podiatry intervention for the prevention of falls in older people. BMJ Open. 2014;4(12).

9. de Negreiros Cabral K, Perracini MR, Soares AT, de Cristo Stein F, Sera CT, Tiedemann A, et al. Effectiveness of a multifactorial falls prevention program in community-dwelling older people when compared to usual care: study protocol for a randomised controlled trial (Prevquedas Brazil). BMC Geriatr. 2013;13:27.

10. Drahota AM, Kward D, Udell JE, Soilemezi D, Ogollah R, Higgins B, et al. Pilot cluster randomised controlled trial of flooring to reduce injuries from falls in wards for older people. Age Ageing. 2013;42(5):633-40.

11. Elley CR, Robertson MC, Kerse NM, Garrett S, McKinlay E, Lawton B, et al. Falls Assessment Clinical Trial (FACT): design, interventions, recruitment strategies and participant characteristics. BMC Public Health. 2007;7:185.

12. Giangregorio LM, Thabane L, Adachi JD, Ashe MC, Bleakney RR, Braun EA, et al. Build better bones with exercise: protocol for a feasibility study of a multicenter randomized controlled trial of 12 months of home exercise in women with a vertebral fracture. Phys Ther. 2014;94(9):1337-52.

13. Gschwind YJ, Eichberg S, Marston HR, Ejupi A, Rosario HD, Kroll M, et al. ICT-based system to predict and prevent falls (iStoppFalls): study protocol for an international multicenter randomized controlled trial. BMC Geriatr. 2014;14(1).

14. Haines TP, Russell T, Brauer SG, Erwin S, Lane P, Urry S, et al. Effectiveness of a video-based exercise programme to reduce falls and improve health-related quality of life among older adults discharged from hospital: a pilot randomized controlled trial. Clin Rehabil. 2009;23(11):973-85.

15. Hawk C, Pfefer MT, Strunk R, Ramcharan M, Uhl N. Feasibility study of short-term effects of chiropractic manipulation on older adults with impaired balance. J Chiropr Med. 2007;6(4):121-31.

16. Henderson EJ, Lord SR, Close JCT, Lawrence AD, Whone A, Ben-Shlomo Y. The ReSPonD trial - rivastigmine to stabilise gait in Parkinson's disease a phase II, randomised, double blind, placebo controlled trial to evaluate the effect of rivastigmine on gait in patients with Parkinson's disease who have fallen. BMC Neurol. 2013;13.

17. Hewitt J, Refshauge KM, Goodall S, Henwood T, Clemson L. Does progressive resistance and balance exercise reduce falls in residential aged care? Randomized controlled trial protocol for the SUNBEAM program. Clin Interv Aging. 2014;9:369-76.

18. Hill AM, Etherton-Beer C, Haines TP. Tailored education for older patients to facilitate engagement in falls prevention strategies after hospital discharge--a pilot randomized controlled trial. PloS One. 2013;8(5):e63450.

19. Liston MB, Alushi L, Bamiou DE, Martin FC, Hopper A, Pavlou M. Feasibility and effect of supplementing a modified OTAGO intervention with multisensory balance exercises in older people who fall: a pilot randomized controlled trial. Clin Rehabil. 2014;28(8):784-93.

20. Merom D, Cumming R, Mathieu E, Anstey KJ, Rissel C, Simpson JM, et al. Can social dancing prevent falls in older adults? a protocol of the Dance, Aging, Cognition, Economics (DAnCE) fall prevention randomised controlled trial. BMC Public Health. 2013;13:477.

21. Mikolaizak AS, Simpson PM, Tiedemann A, Lord SR, Caplan GA, Bendall JC, et al. Intervention to prevent further falls in older people who call an ambulance as a result of a fall: a protocol for the iPREFER randomised controlled trial. BMC Health Serv Res. 2013;13.

22. Morello R, Barker A, Zavarsek S, Watts JJ, Haines T, Hill K, et al. The 6-PACK programme to decrease falls and fall-related injuries in acute hospitals: protocol for an economic evaluation alongside a cluster randomised controlled trial. Inj Prev. 2012;18(2).

23. Morris ME, Martin C, McGinley JL, Huxham FE, Menz HB, Taylor NF, et al. Protocol for a home-based integrated physical therapy program to reduce falls and improve mobility in people with Parkinson's disease. BMC Neurol. 2012;12.

24. Morris ME, Menz HB, McGinley JL, Huxham FE, Murphy AT, Iansek R, et al. Falls and mobility in Parkinson's disease: protocol for a randomised controlled clinical trial. BMC Neurol. 2011;11:93.

25. Parry SW, Deary V, Finch T, Bamford C, Sabin N, McMeekin P, et al. The STRIDE (Strategies to Increase confidence, InDependence and Energy) study: cognitive behavioural therapy-based intervention to reduce fear of falling in older fallers living in the community - study protocol for a randomised controlled trial. Trials. 2014;15:210.

26. Pighills AC, Torgerson DJ, Sheldon TA, Drummond AE, Bland JM. Environmental assessment and modification to prevent falls in older people. J Am Geriatr Soc. 2011;59(1):26-33.

27. Snooks H, Anthony R, Chatters R, Cheung WY, Dale J, Donohoe R, et al. Support and assessment for fall emergency referrals (SAFER 2) research protocol: cluster randomised trial of the clinical and cost effectiveness of new protocols for emergency ambulance paramedics to assess and refer to appropriate community-based care. BMJ Open. 2012;2(6).

28. Snooks H, Cheung WY, Close J, Dale J, Gaze S, Humphreys I, et al. Support and Assessment for Fall Emergency Referrals (SAFER 1) trial protocol. Computerised on-scene decision support for emergency ambulance staff to assess and plan care for o/lder people who have fallen: evaluation of costs and benefits using a pragmatic cluster randomised trial. BMC Emerg Med. 2009;10.

29. Spink MJ, Menz HB, Lord SR. Efficacy of a multifaceted podiatry intervention to improve balance and prevent falls in older people: study protocol for a randomised trial. BMC Geriatr. 2008;8.

30. Tan PJ, Khoo EM, Chinna K, Hill KD, Poi PJH, Tan MP. An individually-tailored multifactorial intervention program for older fallers in a middle-income developing country: Malaysian Falls Assessment and Intervention Trial (MyFAIT). BMC Geriatr. 2014;14(1).

31. Uusi-Rasi K, Kannus P, Karinkanta S, Pasanen M, Patil R, Lamberg-Allardt C, et al. Study protocol for prevention of falls: a randomized controlled trial of effects of vitamin D and exercise on falls prevention. BMC Geriatr. 2012;12.

32. Watts JJ, McGinley JL, Huxham F, Menz HB, Iansek R, Murphy AT, et al. Cost effectiveness of preventing falls and improving mobility in people with Parkinson disease: protocol for an economic evaluation alongside a clinical trial. BMC Geriatr. 2008;8.

Excluded since second report on included trial

33. Ashburn A, Stack E, Ballinger C, Fazakarley L, Fitton C. The circumstances of falls among people with Parkinson's disease and the use of falls diaries to facilitate reporting. Disabil Rehabil. 2008;30(16):1205-12.

34. Dargent-Molina P, Cassou B. Prevention of falls and broken bones in older women: Ossebo random trials. Gerontologie et Societe. 2008;125(2):65-78.

35. Freiberger E, Häberle L, Spirduso WW, Rixt Zijlstra GA. Long-term effects of three multicomponent exercise interventions on physical performance and fall-related psychological outcomes in community-dwelling older adults: a randomized controlled trial. J Am Geriatr Soc. 2012;60(3):437-46.

36. Hill AM, Hoffmann T, Haines TP. Circumstances of falls and falls-related injuries in a cohort of older patients following hospital discharge. Clin Interv Aging. 2013;8:765-74.

37. Kemmler W, Stengel SV, Mayer S, Niedermayer M, Hentschke C, Kalender WA. Effect of whole body vibration on the neuromuscular performance of females 65 years and older: one-year results of the controlled randomized ELVIS study. Z Gerontol Geriatr. 2010;43(2):125-32.

38. Kemmler W, von Stengel S. Exercise frequency, health risk factors, and diseases of the elderly. Arch Phys Med Rehabil. 2013;94(11):2046-53.

39. Kemmler W, von Stengel S, Mayer S, Engelke K, Kalender WA. Exercise effects on risk factors and health care costs in the elderly. Final results of the Senior Fitness and Prevention Study (SEFIP). Deutsche Zeitschrift Fur Sportmedizin. 2010;61(11):264-9.

40. Vind AB, Andersen HE, Pedersen KD, Joergensen T, Schwarz P. Effect of a program of multifactorial fall prevention on health-related quality of life, functional ability, fear of falling and psychological well-being. A randomized controlled trial. Aging Clin Exp Res. 2010;22(3):249-54.

41. von Stengel S, Kemmler W, Mayer S, Engelke K, Klarner A, Kalender WA. Effect of whole body vibration exercise on osteoporotic risk factors. Results of the controlled randomized longitudinal ELVIS study after one year. Deutsche Medizinische Wochenschrift. 2009;134(30):1511-6.

42. Zieschang T, Schwenk M, Becker C, Oster P, Hauer K. Feasibility and accuracy of fall reports in persons with dementia: a prospective observational study. Int Psychogeriatr. 2012;24(4):587-98.

Excluded since non-elderly population

43. Coote S, Hogan N, Franklin S. Falls in people with multiple sclerosis who use a walking aid: prevalence, factors, and effect of strength and balance interventions. Arch Phys Med Rehabil. 2013;94(4):616-21.

Excluded since outcome measurement not falls-related

44. Hawk C, Cambron JA, Pfefer MT. Pilot study of the effect of a limited and extended course of chiropractic care on balance, chronic pain, and dizziness in older adults. J Manipulative Physiol Ther. 2009;32(6):438-47.

45. Hinrichs T, Moschny A, Klaassen-Mielke R, Trampisch U, Thiem U, Platen P. General practitioner advice on physical activity: analyses in a cohort of older primary health care patients (getABI). BMC Fam Pract. 2011;12.

46. McMurdo MET, Sugden J, Argo I, Boyle P, Johnston DW, Sniehotta FF, et al. Do pedometers increase physical activity in sedentary older women? A randomized controlled trial. J Am Geriatr Soc. 2010;58(11):2099-106.

47. Suman S, Myint PK, Clark A, Das P, Ring L, Trepte NJB. Community-based fall assessment compared with hospital-based assessment in community-dwelling older people over 65 at high risk of falling: a randomized study. Aging Clin Exp Res. 2011;23(1):35-41.
