# Supplementary material for: Appraising the uptake and use of recommendations for a common outcome data set for clinical trials: a case study in fall injury prevention
Source: Trials. 2016 Mar 10;17:131. doi: 10.1186/s13063-016-1259-7 (PMC4785736; doi:10.1186/s13063-016-1259-7)
Supplement: Additional file 2: Table S1. — Level of adherence to recommendations within domains by trial. (PDF 229 kb) [file 13063_2016_1259_MOESM2_ESM.pdf]

Additional file 2. Table S1. Level of adherence to recommendations within domains by trial

Author	Year		Falls	Fall-related Injury	Psychological Consequences	Health-related quality of life	Physical activity	Domains reported
**Freiberger**	2007		85%	×	×	×	×	20%
**Luukinen**	2007		54%	25%	×	×		60%
**Vaapio**	2007		×	×	×	50%	×	20%
**Voukelatos**	2007		69%	×	×	×	×	20%
**Elley**	2008		77%	42%	100%	50%		100%
**Rapp**	2008		62%	×	×	×	×	20%
**Shigematsu**	2008		69%	×	×	×	×	20%
**Shigematsu**	2008		69%	×	0%	×	×	40%
**McMurdo**	2009		46%	×	×	50%		60%
**Vind**	2009		85%	42%	×	×	×	40%
**Faes**	2010		54%	×	0%	50%	×	60%
**Fitzharris**	2010		62%	33%	×	×	×	40%
**Kemmler**	2010		54%	42%	×	×	×	40%
**Logan**	2010		85%	42%	50%	×	×	60%
**Smulders**	2010		69%	42%	×	50%		80%
**Goodwin**	2011		46%	×	0%	50%		80%
**Hill**	2011		62%	33%	×	×	×	40%
**Schepens**	2011		×	×	×	×	×	0%
**Spink**	2011		85%	×	0%	50%	×	60%
**Trombetti**	2011		77%	×	×	×	×	20%
**Von Stengel**	2011		23%	17%	×	×	×	40%
**Batchelor**	2012		77%	42%	50%	×		80%
**Perula**	2012		31%	25%	×	×		60%
**Taylor**	2012		77%	×	×	×	×	20%
**Tilson**	2012		77%	42%	×	×	×	40%
**Duque**	2013		31%	×	×	×	×	20%
**Kovacs**	2013		38%	×	×	0%	×	40%
**Kovacs**	2013		62%	×	×	×	×	20%
**Zieschang**	2013		77%	×	×	×		40%
**Ferrer**	2014		69%	33%	×	×	×	40%
**Moller**	2014		54%	25%	×	×	×	40%
**Palvanen**	2014		46%	17%	×	×	×	40%
**Perez-Roz**	2014		31%	33%	×	×		40%
**Van Het Reve**	2014		31%	×	0%	×	×	40%
